# The Protective Effects of α-Mangostin Attenuate Sodium Iodate-Induced Cytotoxicity and Oxidative Injury via Mediating SIRT-3 Inactivation via the PI3K/AKT/PGC-1α Pathway

**DOI:** 10.3390/antiox10121870

**Published:** 2021-11-24

**Authors:** Chen-Ju Chuang, Meilin Wang, Jui-Hsuan Yeh, Tzu-Chun Chen, Shang-Chun Tsou, Yi-Ju Lee, Yuan-Yen Chang, Hui-Wen Lin

**Affiliations:** 1 Emergency Department, Kaohsiung Municipal United Hospital, Kaohsiung 80457, Taiwan; ilovespurs168@gmail.com; 2 Department of Microbiology and Immunology, School of Medicine, Chung Shan Medical University and Clinical Laboratory, Chung Shan Medical University Hospital, Taichung 40201, Taiwan; wml@csmu.edu.tw; 3 Institute of Medicine, Chung Shan Medical University, Taichung 40201, Taiwan; simon855264@gmail.com (J.-H.Y.); cherish4488@gmail.com (T.-C.C.); 4 Department of Nutrition, Chung Shan Medical University, Taichung 40201, Taiwan; eq7bie5d@gmail.com; 5 Department of Pathology, Chung-Shan Medical University, Chung-Shan Medical University Hospital, Taichung 40201, Taiwan; jasmine.lyl@gmail.com; 6 Department of Optometry, Asia University, Taichung 41354, Taiwan; 7 Department of Medical Research, China Medical University Hospital, China Medical University, Taichung 40402, Taiwan

**Keywords:** age-related macular degeneration (AMD), oxidative stress, retinal pigment epithelial (RPE), alpha-mangostin (α-MG), antiapoptotic, antioxidant

## Abstract

It is well known that age-related macular degeneration (AMD) is an irreversible neurodegenerative disease that can cause blindness in the elderly. Oxidative stress-induced retinal pigment epithelial (RPE) cell damage is a part of the pathogenesis of AMD. In this study, we evaluated the protective effect and mechanisms of alpha-mangostin (α-mangostin, α-MG) against NaIO_3_-induced reactive oxygen species (ROS)-dependent toxicity, which activates apoptosis in vivo and in vitro. MTT assay and flow cytometry demonstrated that the pretreatment of ARPE-19 cells with α-MG (0, 3.75, 7.5, and 15 μM) significantly increased cell viability and reduced apoptosis from NaIO_3_-induced oxidative stress in a concentration-dependent manner, which was achieved by the inhibition of Bax, cleaved PARP-1, cleaved caspase-3 protein expression, and enhancement of Bcl-2 protein. Furthermore, pre-incubation of ARPE-19 cells with α-MG markedly inhibited the intracellular ROS and extracellular H_2_O_2_ generation via blocking of the abnormal enzyme activities of superoxide dismutase (SOD), the downregulated levels of catalase (CAT), and the endogenous antioxidant, glutathione (GSH), which were regulated by decreasing PI3K-AKT-PGC-1α-STRT-3 signaling in ARPE-19 cells. In addition, our in vivo results indicated that α-MG improved retinal deformation and increased the thickness of both the outer nuclear layer and inner nuclear layer by inhibiting the expression of cleaved caspase-3 protein. Taken together, our results suggest that α-MG effectively protects human ARPE-19 cells from NaIO_3_-induced oxidative damage via antiapoptotic and antioxidant effects.

## 1. Introduction

Retinal pigmented epithelial cells (RPEs) are located between photoreceptor cells (cone and rod cells) and Bruch’s membrane [1]. RPEs are highly metabolically active and sensitive to oxidative stress when the retina is exposed to many reactive oxygen species (ROS) [2]. However, abnormal RPEs are regarded as playing an important role in age-related macular degeneration (AMD), a disease that results in irreversible center vision loss in the elderly population. This disease can generally be divided into two categories: dry AMD and wet AMD. The former is characterized by accumulation of drusen in the retina, thickening of Bruch’s membrane, RPE cell and photoreceptor dysfunction, and severe abnormal neovascularization (angiogenesis), which grows into the central region of the retina, leading to retinal exudation, hemorrhage, and, eventually, serious impairment of vision [3,4]. At present, due to the clinical application of anti-vascular endothelial growth factor (VEGF) agents, wet AMD can be effectively controlled. However, the underlying mechanism of the pathology of dry AMD is still largely unknown. Therefore, there are currently no effective treatment options for dry AMD.

In cellular systems, the generation of reactive oxygen species (ROS) can cause cell damage and affect the structure and function of membrane lipids, proteins, and nucleic acids [5]. ROS is considered to be an important mechanism that leads to a variety of neurodegenerative diseases, such as AMD. Previous studies have demonstrated that inhibiting ROS-induced RPE cell damage may inhibit AMD progression [6,7,8]. Catalase, superoxide dismutase (SOD), and glutathione (GSH) are major enzymes that protect RPE cells through increased expression by effectively scavenging ROS and attenuating oxidative damage [9].

PGC-1α, PGC-1β, and PRC (PGC-1-related coactivator) belong to the peroxisome proliferator-activated receptor gamma coactivator-1 (PGC-1) family. They can regulate the biogenesis and respiratory function of mitochondria as well as targeting the mitochondrial antioxidant defense system [10]. The role of PGC-1α is to serve as a switch between mitochondrial biogenesis and oxidative damage by controlling the mitochondrial levels of ROS. Loss-of-function studies of PGC-1α have shown bursts of ROS and an increase in mitochondrial damage and degradation, whereas gain-of-function enhanced mitochondrial biogenesis and the expression of mitochondrial antioxidant defense system-related genes, such as SOD2 (superoxide dismutase 2) and TRX1 (thioredoxin) [10]. However, both pathways, mitochondrial biogenesis and ROS control, strive for the preservation of mitochondrial homeostasis. Post-translational modification of the energy sensors, AMPK (AMP-activated protein kinase), and SIRT-1/3 (sirtuin-1/3), which induce autophagy, are known to regulate PGC-1α activities [11]. In RPE cells, PGC-1α has been shown to drive mitochondrial biogenesis and to activate the antioxidant defense system [8,12]. Recent studies have reported that SOD2 is a major downstream signal of SIRT-3-mediated mitochondrial O2^•−^ reduction, and deacetylation of SOD2 by the SIRT-3 pathway regulates SOD2 enzymatic activity [13]. We therefore hypothesized that NaIO_3_ might induce mitochondrial ROS in RPE cells and that scavenging mitochondrial ROS could be an effective strategy for preventing NaIO_3_-induced retinal toxicity.

Mangosteen (known as the “Queen of Fruit”), which is edible and also known to have medicinal properties, is generally cultivated in Southeast Asia [14]. The major bioactive secondary metabolites of mangosteen have been found to be xanthone derivatives, including α, β, and γ mangostin. Alpha-mangostin (α-MG) has antiinflammatory [15,16], antibacterial [17,18], antioxidant [19], anticancer [20,21], and cardioprotective [22] activities both in vitro and in vivo. α-MG possesses strong antioxidant activity that has gradually been confirmed in recent years. The researchers found that α-MG displayed the ability to scavenge ROS in each dose [23]. Márquez-Valadez and coworkers (2009) found that α-MG could be considered an accurate antioxidant/neuroprotective tool to be employed in further paradigms of neuronal cell damage [24]. Although α-MG has shown a variety of antioxidant and protective properties, its effects on retina health have not yet been clarified. In this study, we will evaluate whether α-MG may reduce or prevent NaIO_3_-induced retinal damage both in vitro and in vivo.

## 2. Materials and Methods

### 2.1. Cell Culture

The ARPE-19 cell line was purchased from the American Type Culture Collection (Manassas, VA, USA). The cells were cultured in Dulbecco’s modified Eagle medium (DMEM) supplemented with 10% heat inactivated fetal bovine serum (FBS) (Gibco; Thermo Fisher Scientific, Inc., Waltham, MA, USA), 100 μg/mL streptomycin and 100 U/mL penicillin (CSPC Pharmaceutical Group Ltd., Shijiazhuang, China) at 37 °C in an atmosphere containing 5% CO_2_. The cells were passaged every three days once grown to ~90% confluence.

### 2.2. Cell Viability Assay

ARPE-19 cells (1.5 × 10^5^ cells/well) were seeded into 24-well plates at 1 mL volume and incubated at 37 °C for 24 h. The culture medium was subsequently replaced by a medium containing various doses of α-MG (0, 3.75, 7.5, and 15 μM) alone or with a co-treatment of NaIO_3_ for 24 h. For the cell viability assay condition and procedure, refer to [25].

### 2.3. Measurement of Intracellular ROS Production

The effect of different concentrations of α-MG (0, 3.75, 7.5, and 15 μM) on NaIO_3_-induced intracellular ROS generation in ARPE-19 cells was further confirmed by flow cytometry (BD Biosciences, San Jose, CA, USA), using DCFH-DA fluorogenic dye as a probe. Data analysis was performed using CellQuest.

### 2.4. Measurement of Mitochondrial Damage

For the analysis of mitochondrial status, the cells were incubated with JC-1 dye (2 μg/mL; Cayman Chemical, Ann Arbor, MN, USA) for 50 min. Two μL of Hoechst33342, a DNA-specific fluorescent dye, was added, and the cells were incubated in darkness at 37 °C for 10 min. For the conditions and procedure, refer to [25].

### 2.5. Measurements of Antioxidative Capacities and Extracellular H_2_O_2_ Production

The effects of different concentrations of α-MG (pretreatment 0, 3.75, 7.5, 15 μM) on the NaIO_3_-induced antioxidative capacities and H_2_O_2_ generation in ARPE-19 cells were further confirmed by ELISA kits. The activities of superoxide dismutase (SOD), catalase (CAT), and reduced glutathione (GSH) were analyzed using ELISA kits from Cayman according to the manufacturer’s instructions (Cat. 706002, 707002, and 703002, Cayman, Ann Arbor, MI, USA). The extracellular H_2_O_2_ production was determined using a Biovision assay kit (Biovision Research Products, Milpitas, CA, USA) following the manufacturer’s instructions.

### 2.6. Western Blotting 

The ARPE-19 cells were pretreated under different conditions for 24 h and, after the incubation of cell lysates with different primary antibodies including Bcl2 (ab182858) purchased from abcam (Cambridge, UK), Bax (2D2), cleaved caspase-3 (31A1067), cleaved PARP-1 (194C1439), PI3K-p110 (H-239), p-AKT (B-5), PGC-1α (D-5), SIRT-3, and GAPDH (6C5) purchased from Santa Cruz (Santa Cruz, CA, USA), and SIRT-3 (D22A3) purchased from Cell Signaling (Beverly, MA, USA) primary antibodies, immunoblotting was performed, as described previously in [16].

### 2.7. Animal Model

Eight-week-old BABL/c mice were purchased from the Asia University Animal Care and Use Committee (IACVC No. 107-a51a-20, 01 August 2019) and housed in standard cages with a 12-h light–dark cycle. The mice were randomly divided into three groups with each group containing six mice:

1. Mock group: Animals pretreated with an intraperitoneal (IP) injection of PBS and then a single intravenous (IV) injection of PBS.
2. Vehicle-treated group: Animals pretreated with an IP injection of PBS and then a single IV injection of 40 mg/kg NaIO_3_ [26].
3. Experimental group: Animals pretreated with an IP injection of 20 mg/kg α-MG and then a single IV injection of 40 mg/kg NaIO_3_ [27].

### 2.8. Histology and Immunohistochemistry

The mice were sacrificed, and both eyes were surgically collected and applied to the Davidson’s solution (containing 10% formalin, 10% glacial acetic acid, and 4% formaldehyde) to fix the specimen for three days [28]. Eyeball tissues were embedded in paraffin before sectioned at 5 μm-thick and stained with hematoxylin and eosin (H&E) to capture the tissue section image via optical microscope (Olympus Optical, Tokyo, Japan). Different retinal thickness, i.e., whole retina, inner nuclear layer (INL), and outer nuclear layer (ONL) were randomly measured 6 times from the inferior and superior hemiretina in the range of 600–900 μm on nasal and temporal side of the optic nerve. 

Besides the expression of caspase-3 in mice retina evaluated by Image J Immunohistochemistry Toolbox (National Institute of Health, Starkville, MD, USA), the tissue sections were incubated with cleaved caspase-3 (1:100, Cell Signaling Technology, Danvers, MA, USA) antibody in BondMax automated slide staining system (Vision BioSystems Ltd., Newcastle Upon Tyne, UK) and the tissue section image screenshotted.

### 2.9. Retinal Imaging

Optical coherence tomography (OCT) was performed using RTVue XR Avanti with AngioVue (Optovue Inc, Fremont, CA, USA). Briefly, OCT of a certain region of the retina was performed repeatedly, and the resultant scans were examined for changes [27].

### 2.10. Statistical Analysis

All grouping and subject selection in this study were decided completely at random. All data were analyzed in SPSS software. Multiple group comparisons were performed using one-way ANOVA followed by least significant difference (LSD) post hoc test. *p* < 0.05 indicated statistical significance for all tests. The values of the results were representative in terms of the mean ± standard deviation (SD).

## 3. Results

### 3.1. α-Mangostin (α-MG) Protects ARPE-19 from NaIO_3_-Induced Damage

**Figure 1:**
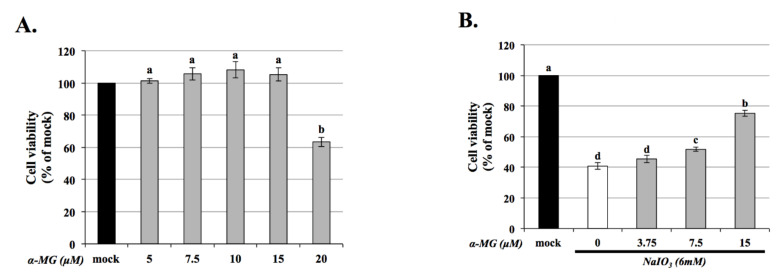
α-MG attenuates NaIO_3_-induced cytotoxicity in ARPE-19 cells. (**A**) ARPE-19 cells were treated with different concentrations of α-MG (5, 7.5, 10, 15, and 20 μM) for 24 h. (**B**) Effect of α-MG against NaIO_3_-induced cytotoxicity in ARPE-19 cells. Before being NaIO_3_ (6 mM) treated, three different concentrations of α-MG (3.75, 7.5, and 15 μM) were pretreated with ARPE-19 cells for 1.5 h, and, after 24 h, the cell viability was measured using a CCK-8 assay. Data were expressed by mean ± SD (*n* = 3). Values without a common superscript letter are significantly different (*p* < 0.05).

To confirm the cytotoxicity of α-MG on ARPE-19, the cells were pretreated with α-MG (0, 3.75, 7.5, and 15 μM) alone or with a co-treatment of NaIO_3_. After 24 h, the cell viability of ARPE-19 was assessed using an MTT assay. The cell survival rate was unaffected following the treatment of cells with < 15 μM α-MG (Figure 1A). However, treatment with 20 μM α-MG significantly reduced the cell viability, with the effects being significantly different from the untreated controls (0 μM). For this reason, <15 μM α-MG was used for all subsequent experiments. 

We also evaluated whether α-MG treatment could reduce ARPE-19 cell damage induced by NaIO_3_. Before being treated with NaIO_3_ (6 mM), the ARPE-19 cells were pretreated with α-MG (3.75, 7.5, and 15 μM) for 1.5 h. Based on the results, significantly increased protective effects were found on cell death, compared with the group treated with NaIO_3_ only (Figure 1B). 

### 3.2. Treatment with α-MG Suppressed NaIO_3_-Induced Mitochondrial Dysfunction

**Figure 2:**
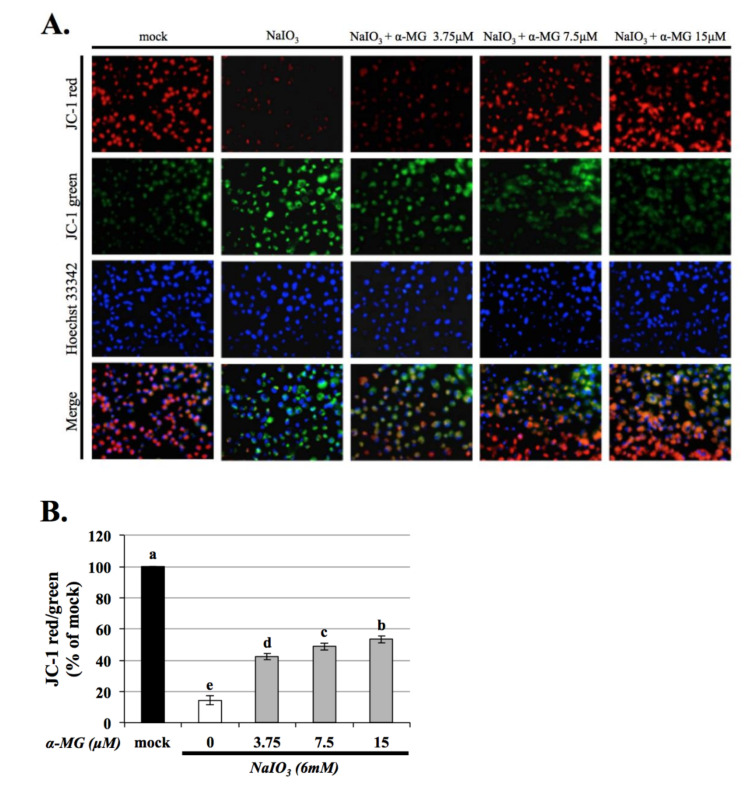
α-MG suppressed NaIO_3_-induced mitochondrial dysfunction in ARPE-19 cells. (**A**) Representative images of staining with JC-1dye. The green areas represent the JC-1 monomer, which indicates impaired mitochondria, and the red areas represent the JC-1 polymer, which indicates healthy mitochondria. (**B**) Quantitative data obtained from the JC-1 staining assay. Data were expressed by mean ± SD (*n* = 4). Values without a common superscript letter are significantly different (*p* < 0.05).

The JC-1 staining assay is used to evaluate the function of mitochondria [29]. JC-1 monomers emit green fluorescence and indicate damaged mitochondria, while JC-1 polymers emit red fluorescence and indicate healthy mitochondria. The treatment of ARPE-19 cells with NaIO_3_ for 24 h caused the accumulation of JC-1 monomers in the mitochondria, which was reduced in a dose-dependent manner following treatment with various concentrations of α-MG (0, 3.75, 7.5, and 15 μM). (Figure 2A). Quantitative data revealed that the green/red fluorescence ratio and NaIO_3_-induced mitochondrial damage increased following treatment with NaIO_3_ and improved following treatments with α-MG (Figure 2B). The results indicated that NaIO_3_-induced mitochondrial dysfunction was improved via the inhibition of mitochondrial fission, but α-MG can alleviate this appearance.

### 3.3. α-MG Reduces NaIO_3_ Induced Intracellular ROS and Extracellular H_2_O_2_ Generation in ARPE-19 Cells

**Figure 3:**
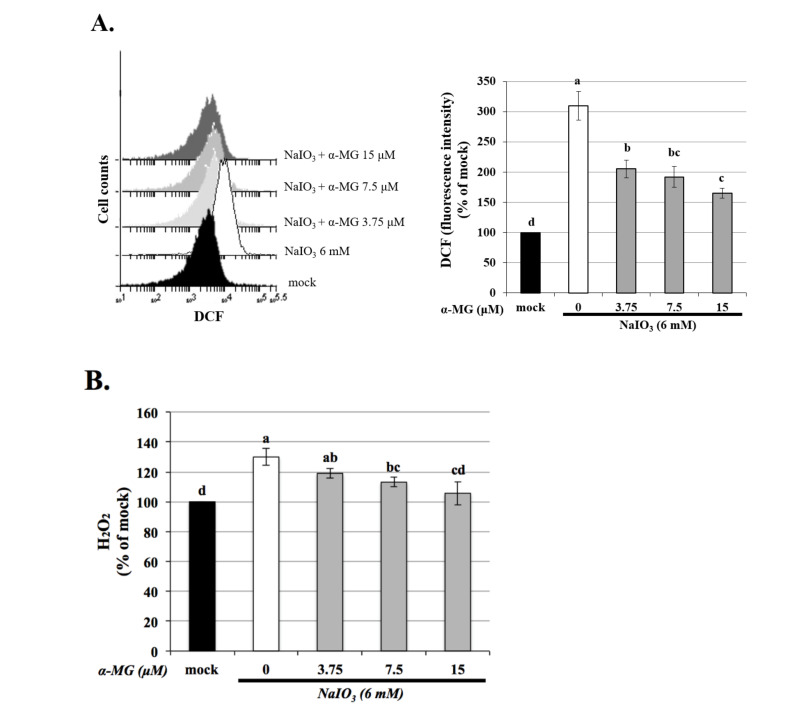
NaIO_3_-induced intracellular ROS and extracellular H_2_O_2_ production in ARPE-19 cells was decreased by α-MG. Before NaIO_3_ (6 mM) treatment, three different concentrations of α-MG (3.75, 7.5, and 15 μM) were pretreated to ARPE-19 cells for 1.5 h. Assessing the intracellular ROS (**A**) and H_2_O_2_ (**B**) generation level were detected by flowcytometry and commercial assay kits, respectively. Data were expressed by mean ± SD (*n* = 3). Values without a common superscript letter are significantly different (*p* < 0.05).

Previous research demonstrated that mitochondrial dysfunction induces the production of intracellular ROS [30]. We measured the levels of intracellular ROS using DCFH-DA (an intracellular ROS probe) and analyzed through flow cytometry. In agreement with the previous reports [8,25], treatment of the cultured cells with 6 mM of NaIO_3_ caused approximately 60% cell death (Figure 1B) and an approximately 200% increase in intracellular ROS production (Figure 3A) respectively. Compared with the NaIO_3_-treated group, pretreatment with α-MG attenuated the NaIO_3_-induced intracellular ROS levels in the ARPE-19 cells (Figure 3A). We then measured the level of extracellular H_2_O_2_ using a commercial kit, and the results showed that the increased expression of H_2_O_2_ in the NaIO_3_-induced group was significantly eliminated after treatment with α-MG (Figure 3B). These results established α-MG as an effective antioxidant against NaIO_3_-induced oxidative stress by reducing ROS.

### 3.4. α-MG Improves NaIO_3_-Induced Decreasing Antioxidant Status in ARPE-19 Cells

**Figure 4:**
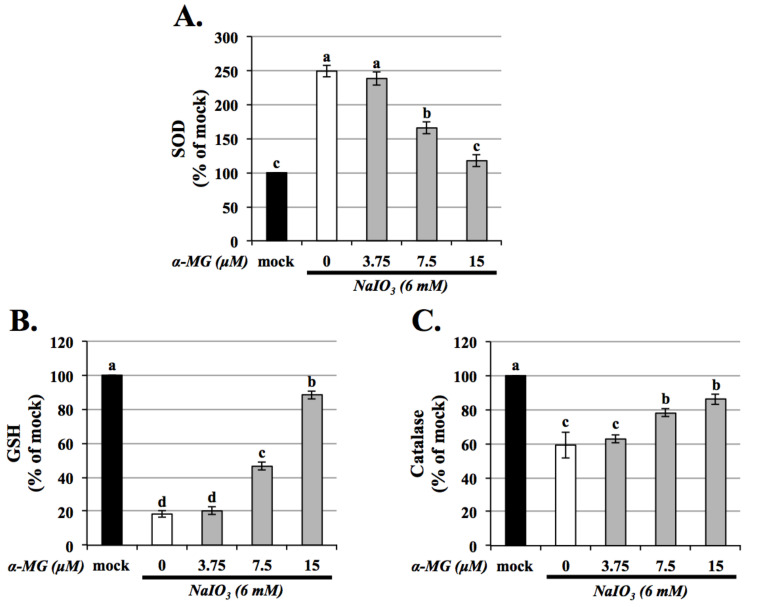
Effects of alpha-mangostin on the expression of SOD, GSH, and catalase levels in NaIO_3_-induced ARPE-19 cells. Before NaIO_3_ (6 mM) treatment, three different concentrations of α-MG (3.75, 7.5, and 15 μM) were pretreated to ARPE-19 cells for 1.5 h. The cell lysates were collected to assess the activities of SOD (**A**), glutathione (GSH) (**B**), and catalase (**C**) by commercial assay kits. Data were expressed by mean ± SD (*n* = 3). Values without a common superscript letter are significantly different (*p* < 0.05).

In mammals, the cellular ROS generation major occurs in mitochondria, which are also considered as the center of cellular bioenergetics. SOD2 is a mitochondrial antioxidant that helps to eliminate O_2_^●−^ and induce the production of H_2_O_2_. However, if H_2_O_2_ cannot be metabolized, ROS will be produced. Antioxidant enzymes play a major role in ROS scavenging, and changes in their expression or/and activity are reported to be associated with AMD. As shown in Figure 4, the expressions of SOD activity were increased, but catalase and GSH were decreased in the NaIO_3_-induced group. α-MG (7.5 and 15 μM) treatment significantly reversed the reduced levels of catalase and GSH and the increased level of SOD, indicating that α-MG could modulate the activity of antioxidants. These results suggested that α-MG decreased NaIO_3_-induced oxidative stress by increasing intracellular antioxidant enzyme activity. 

### 3.5. α-MG Protects NaIO_3_-Induced Apoptosis in ARPE-19 Cells

**Figure 5:**
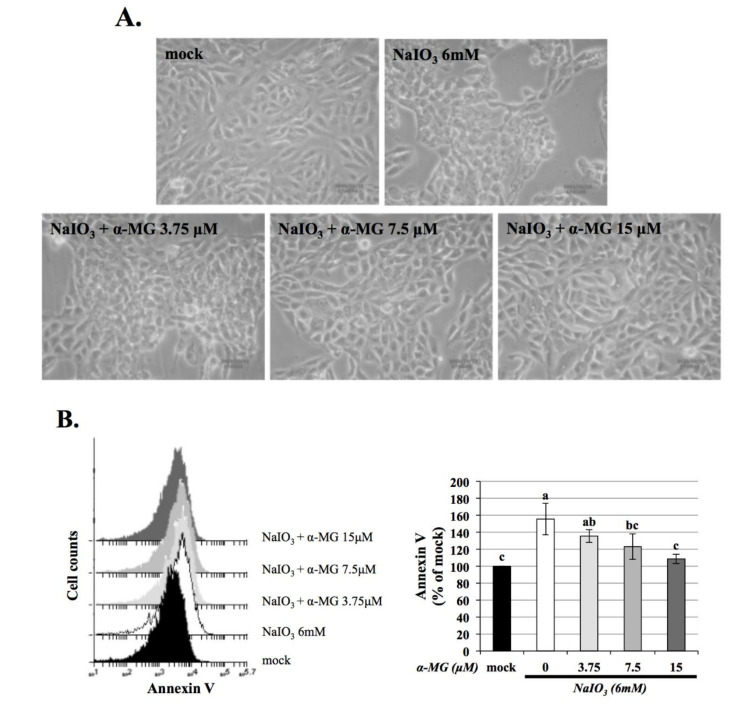
α-MG suppressed NaIO_3_-induced ARPE-19 cell apoptosis. (**A**) Morphological images of ARPE-19 cells following different treatment. (**B**) ARPE-19 cells were pretreated with the indicated concentrations of α-MG (0, 3.75, 7.5, and 15 μM) for 1.5 h, followed by NaIO_3_ (6 mM) administration for 24 h. Apoptosis was measured by flow cytometry using Annexin-V staining. Data were expressed by mean ± SD (*n* = 3). Values without a common superscript letter are significantly different (*p* < 0.05).

Oxidative stress can induce cell apoptosis. To further investigate this protective effect of α-MG on the cell viability of ARPE-19 cells, the morphological change and apoptosis rate of ARPE-19 cells, incubated with α-MG (3.75, 7.5, and 15 μM) for 1.5 h, and then exposed to 6 mM NaIO_3_ for 24 h, were determined under a microscope (Figure 5A) and by flow cytometry (Figure 5B), respectively. Consistently, exposure to 6 mM NaIO_3_ led to a significantly higher rate of total apoptosis compared with that of the control group; however, pretreatment of ARPE-19 cells with α-MG at concentrations of 3.75, 7.5, and 15 μM before NaIO_3_ exposure significantly decreased NaIO_3_-induced cell apoptosis. These results confirmed that α-MG inhibiting ROS levels could rescue NaIO_3_-induced retinal toxicity by apoptosis.

### 3.6. Effect of α-MG on Apoptotic-Related Protein Expression in NaIO_3_-Treated ARPE-19 Cells 

**Figure 6:**
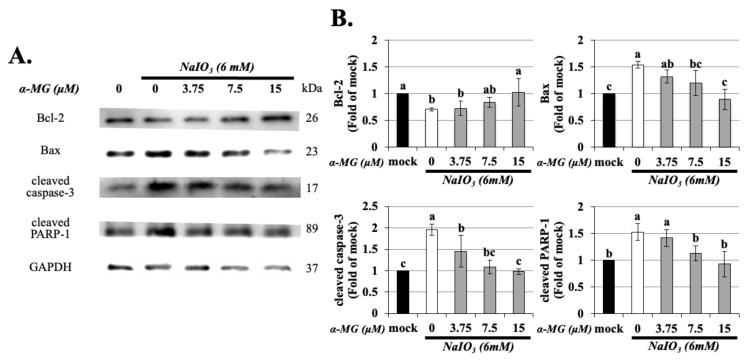
α-MG reduced the expressions of Bcl-2, Bax, cleaved caspase-3, and cleaved PARP-1 in NaIO_3_-treated ARPE-19 cells. ARPE-19 cells were either untreated (mock) or treated with α-MG (0, 3.75, 7.5, and 15 μM) followed by NaIO_3_ (6 mM). Total protein from the ARPE-19 cells was extracted for the measurement of Bcl-2, Bax, cleaved caspase-3, and cleaved PARP-1 expression using Western blot analysis (**A**). The quantified expressions of (**B**), as mean ± SD (*n* = 3, 3 individual set experiments of groups). GAPDH density was used as internal control to normalize all proteins expression levels. Values without a common superscript letter are significantly different (*p* < 0.05).

To confirm the protective effect of α-MG against NaIO_3_-induced apoptosis in ARPE-19 cells, we further investigated the effects of α-MG on the expression of apoptosis-related molecules, i.e., Bcl-2, Bax, cleaved PARP-1, and cleaved caspase-3 by Western blotting (Figure 6). Compared with normal control cells, exposure to NaIO_3_ induced lower levels of Bcl-2 and higher levels of Bax, cleaved PARP-1, and cleaved caspase-3. However, antioxidants of ARPE-19 cells with α-MG (3.75, 7.5 and 15 μM) for 1.5 h dose-dependently reversed this situation, as shown by the decreased expression of Bax, cleaved PARP-1, and cleaved caspase-3, as well as the increased expression of Bcl-2, which was statistically significant relative to those in NaIO_3_ group. 

### 3.7. α-MG Downregulates PGC-1α and SIRT-3 Expression through the Inhibition of PI3K/AKT Signaling Pathway in NaIO_3_-Treated ARPE-19 Cells

**Figure 7:**
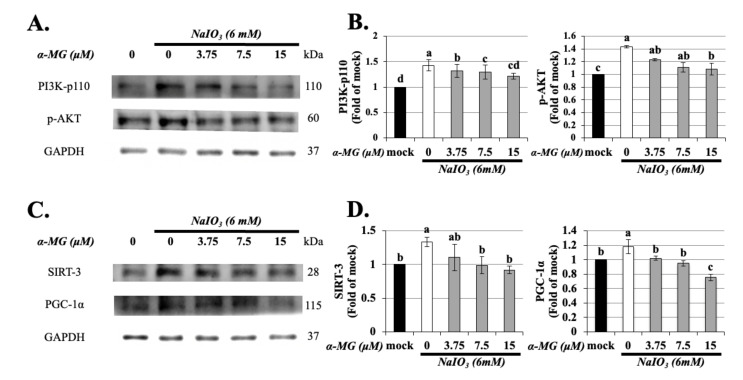
α-MG reduced the expressions of PI3K, p-AKT, SIRT-3, and PGC-1α in NaIO_3_-treated ARPE-19 cells. ARPE-19 cells were either untreated (mock) or treated with α-MG (0, 3.75, 7.5, and 15 μM) followed by NaIO_3_ (6 mM). Total protein from the ARPE-19 cells was extracted for the measurement of PI3K, p-AKT, SIRT-3, and PGC-1α expressions using Western blot analysis (**A**,**C**). The quantified expressions of (**B**,**D**), as mean ± SD (*n* = 3, 3 individual set experiments of groups). GAPDH density was used as internal control to normalize all proteins expression levels. Values without a common superscript letter are significantly different (*p* < 0.05).

Sirtuin-3 (SIRT-3) is crucial for controlling mitochondrial metabolism and homeostasis and protects cells from death under conditions of stress [31]. Du et al. (2019) demonstrated that SIRT-3 was implicated in the effect of H_2_O_2_-induced RPE cell oxidative stress, autophagy, and apoptosis [32]. In this study, we assessed whether SIRT-3 was required for protection from ROS production in RPE cells during NaIO_3_ treatment. To explore the protective mechanisms of α-MG, the PI3K/AKT signaling pathway and the expression of PGC-1α/ SIRT-3 were detected with western blot. The results suggested that the expression of PI3K and phosphorylation of AKT on ARPE-19 cells were significantly increased after 24 h of NaIO_3_ treatment compared with the normal control group, which had been downregulated by α-MG (Figure 7A,B). Simultaneously, as shown in Figure 7C,D, a statistically significant increase in PGC-1α and SIRT-3 protein expression was shown in the NaIO_3_ group. Along with the result of α-MG on the depression of PGC-1α and SIRT-3 protein levels, we found that α-MG protected RPE cells against NaIO_3_-induced oxidative damage by reducing the SIRT-3 expression mediated by the PI3K/AKT/ PGC-1α signaling pathway.

### 3.8. α-MG Inhibition Preserves the Physiological Function of the Retina in Mice following NaIO_3_ Treatment

**Figure 8:**
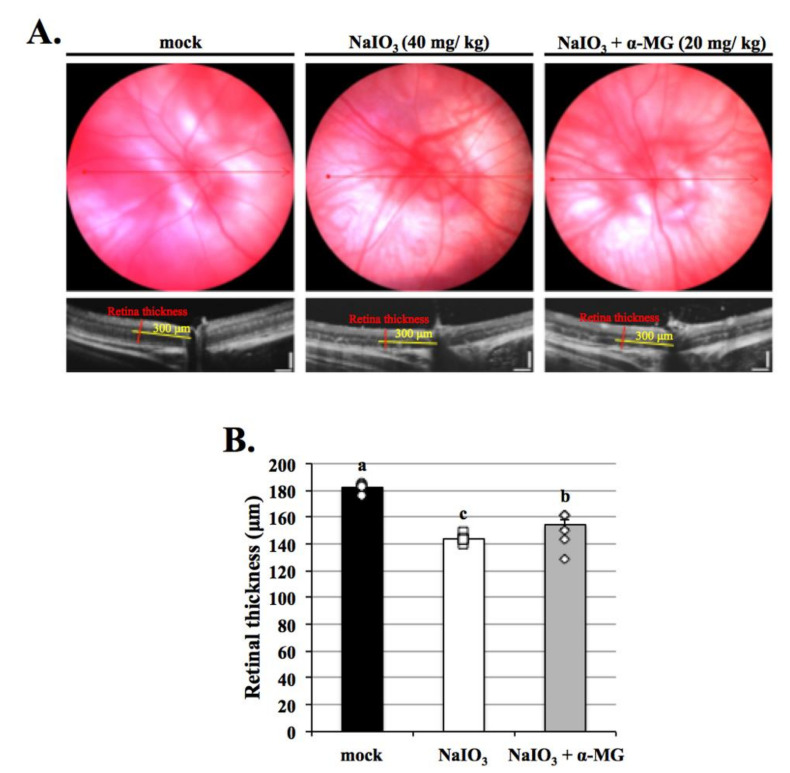
Effects of α-MG on the thinning of retinas in vivo. (**A**) Optical coherence tomography measurement of the effects of α-MG on retinas treated with NaIO_3_ in mice. (**B**) There was significantly increased thinning of retinas in the NaIO_3_ group compared with the mock group; however, α-MG significantly inhibited thinning of retinas after treatment of NaIO_3_ on day seven, compared with the NaIO_3_ groups. Data were measured by mean ± SD (*n* = 6). Values without a common superscript letter are significantly different (*p* < 0.05).

To validate the role of α-MG in the pathogenesis of AMD in vivo, NaIO_3_-injected mice were used as an animal mode [8,25]. We then performed optical coherence tomography (OCT), hematoxylin, and eosin staining to assess geographic atrophy in mice. Optical coherence tomography was performed to detect the effects of α-MG on the retina injuries induced by NaIO_3_. The results showed that α-MG, at the end of the detection points, significantly decreased the thinning of the retina caused by NaIO_3_ treatment (Figure 8A,B).

**Figure 9:**
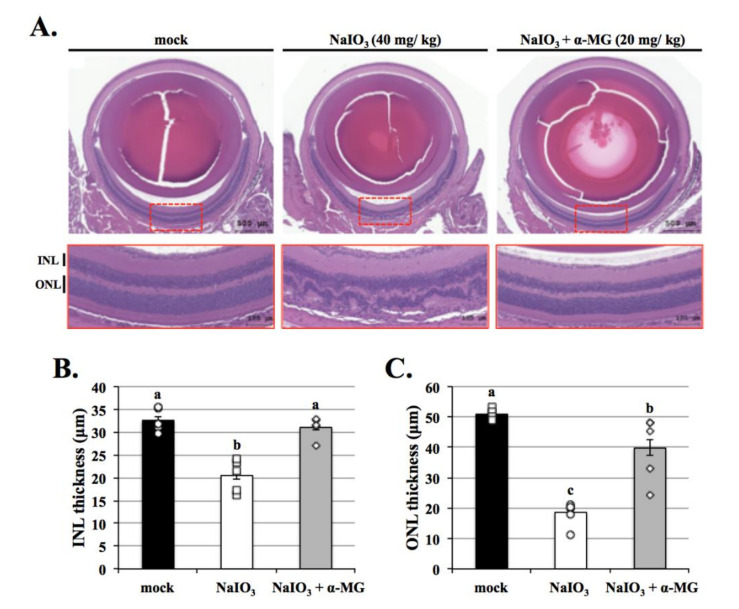
Protective effects of α-MG on retinal degeneration in NaIO_3_-treated mice. (**A**) Different three retinal sections (H&E staining) from three groups were captured of the tissue section image via optical microscope (Olympus Optical, Tokyo, Japan). RPE, retinal pigment epithelium; IS-OS, inner and outer segment of photoreceptor; ONL, outer nuclear layer; INL, inner nuclear layer. Scale bar = 50 μm. There were marked six different positions to measure (**B**) INL and (**C**) ONL thickness were randomly measured 6 times between the range of 600–900 μm on nasal and temporal side of the optic nerve to take the average. Data were expressed by mean ± SD (*n* = 6). Values without a common superscript letter are significantly different (*p* < 0.05).

To further prove this, hematoxylin and eosin (H&E) staining was performed to detect the effects of α-MG on the pathological changes of retina after NaIO_3_ administration. Alterations in retinal morphology began to appear seven days after NaIO_3_ injection in mice, with a reduction in the outer nuclear layer (ONL) and inner nuclear layer (INL) (Figure 9). In contrast, α-MG moderated the alterations in retinal morphology by preserving the ONL and INL thickness of the retina after NaIO_3_ administration. As shown in the graph of H&E staining, compared with the mock group, the thickness of INL and ONL were decreased by about 10–30 μm after the induction of NaIO_3_, which could be reversed by about 10–20 μm with α-MG pretreatment. Collectively, our data show that α-MG mediates retinal degeneration in a dry AMD mouse model.

### 3.9. α-MG Reduced Apoptosis on Retinal Apoptosis Induced by NaIO_3_ in Mice following NaIO_3_ Treatment

**Figure 10:**
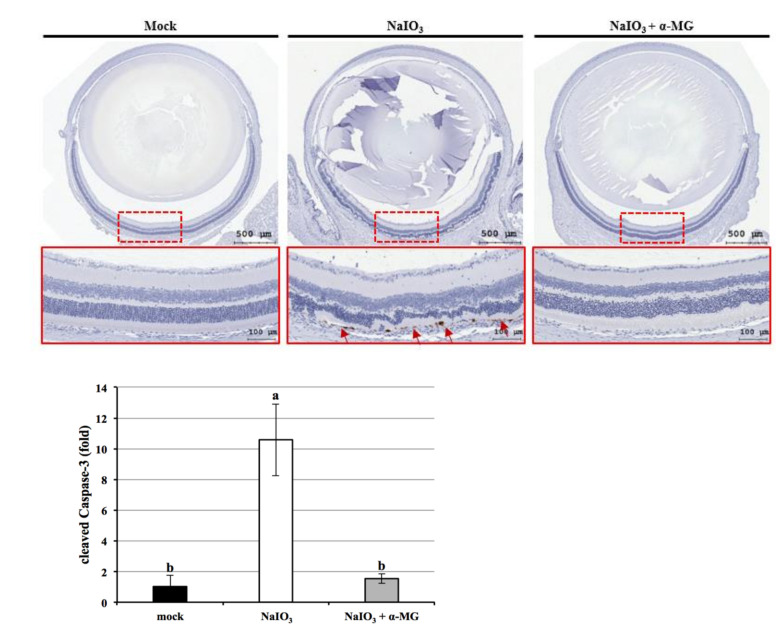
Effect of α-MG on NaIO_3_-induced retinal cell damage via apoptosis in mice. All retinal sections in three study groups were collected after seven days of NaIO_3_ treatment and stained with cleaved caspase-3 by immunohistochemical. Red arrows represent the performance of cleaved caspase-3 (brown) in retinal pigment epithelial (RPE) layers. Compared with mock and experimental (NaIO_3_ + α-MG) groups, the NaIO_3_ group expressed a significantly large amount of cleaved caspase-3. Data were expressed by mean ± SD (*n* = 6). Values without a common superscript letter are significantly different (*p* < 0.05).

Previous research has shown through in vitro studies that α-MG inhibiting ROS levels could rescue NaIO_3_-induced retinal toxicity by apoptosis. Therefore, we further explored whether α-MG could inhibit the protective effect of NaIO_3_-induced retinal cell damage by regulation cleaved caspase-3. We performed immunohistochemical staining on the cleaved caspase-3 after seven days of treated mice. The results showed that in the mock and α-MG groups, the expression level of cleaved caspase-3 was significantly lower in the NaIO_3_-treated group (Figure 10).

## 4. Discussion

NaIO_3_, as a kind of stable oxidant, has been shown to effectively induce retinal degeneration; this model is widely used because of its reproducibility and controllable degree of retinal damage [33]. NaIO_3_-induced cell death in RPE cells is a valuable in vitro model of AMD. Although many studies using this model demonstrate the death features and molecular events underlying the oxidative stress-mediated cellular responses mimicking the pathogenesis of AMD, it remains unclear regarding ROS-mediated signaling pathways, mitochondrial function, autophagy, and mitophagy, which are integrated to control cell viability in RPE [8,25]. 

Excessive mitochondrial ROS are known to induce apoptosis. It is well known that the activity of GSH, SOD, and catalase protects cells against ROS-induced oxidative damage [34]. As shown in Figure 4, the expressions of antioxidants except SOD were dramatically decreased in the NaIO_3_-induced group. α-MG treatment significantly reversed the reduced levels of catalase and GSH and the increased level of SOD, indicating that α-MG could modulate the activity of antioxidants. Previous research reported that NaIO_3_ induced cytosolic ROS production, but not mitochondrial ROS production; furthermore, it activated ERK, p38, JNK, and protein kinase B (AKT) signaling pathways [35]. Consistently, in our study, the cytosolic ROS production in the ARPE-19 cells was demonstrated under the NaIO_3_-induced retinal degeneration, which was detected as intracellular ROS by DCFH-DA fluorescent probes (Figure 3) and extracellular H_2_O_2_ production. Additionally, we found that the induction of NaIO_3_ also promoted mitochondrial damage via the relative intensities of green/red JC-1 fluorescence (Figure 2). These results indicate that α-MG could protect cells from NaIO_3_-mediated ROS injury by maintaining ROS levels and mitochondrial disruption. In addition, in vivo, we found that α-MG significantly retarded the atrophy of NaIO_3_-induced thickness of the total retina, INL, and ONL of NaIO_3_-treated mice at seven days (Figure 9).

It is well known that PGC-1α participates in the regulation of mitochondrial function and redox state [36]. PGC-1α has been reported to be the upstream target gene of SIRT-1/3, indicating that PGC-1α can strongly stimulate SIRT-3 gene expression [37,38]. SIRT-3, a member of the sirtuin family, is localized in the mitochondria and plays pivotal roles in oxidative pathways by targeting several enzymes [39]. SIRT-3 plays important roles in the inhibition of oxidative stress-induced retinal cell death in early diabetic rats [40]. We observed that α-MG treatment decreased PGC-1α and SIRT-3 expression in NaIO_3_-induced RPE cells (Figure 7C,D). With this result, we prove that PGC-1α and SIRT-3 are related to the effects of NaIO_3_-induced oxidative stress and apoptosis in RPE cells in vitro, but the treatment of α-MG can attenuate this appearance. Consequently, this study showed the capacity of α-MG on NaIO_3_-induced RPE cell oxidative stress and apoptosis and the importance of SIRT-3 on mitochondrial ROS regulation in ARPE-19 cells. Finally, we confirmed by the activated PI3K/AKT-PGC-1α signaling pathway that the expression of SIRT-3 was involved in the action of α-MG on RPE cell apoptosis.

In our previous studies, Chang et al. (2021) and Chan et al. (2019), we reported that NaIO_3_-induced PI3K/AKT activation was partially dependent on ROS production, eventually leading to cell death by apoptosis [25,41]. These results indicate that NaIO_3_ induces apoptosis by increasing the cleaved form of caspase-3, PARP-1, and the Bax/Bcl-2 ratio in RPE cells. In addition, we found that α-MG significantly reduced the production of NaIO_3_-induced cleaved caspase-3 expression by in vivo (Figure 10).

**Figure 11:**
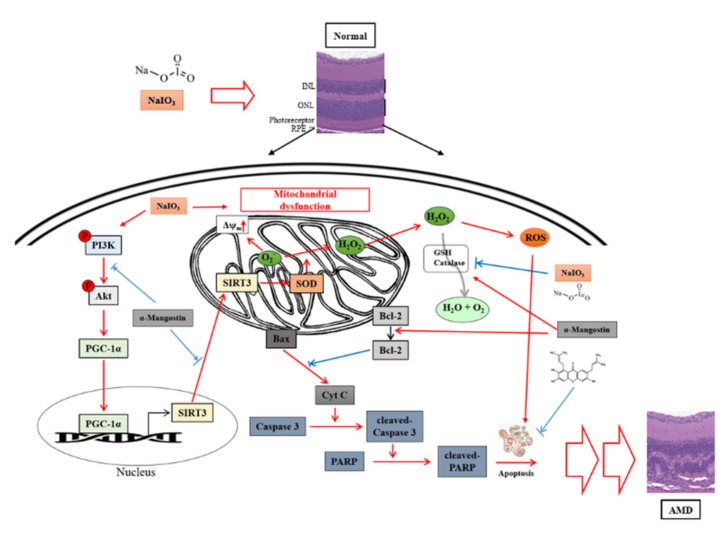
The possible role of α-MG on attenuating sodium iodate-induced RPE cells cytotoxicity and retinal oxidative injury.

In this study, we found that α-MG inhibited NaIO_3_-induced cell apoptosis by mediating PI3K/AKT inactivation (Figure 7), thereby downregulating the expressions of Bax, cleaved caspase-3, and cleaved PARP and upregulating Bcl-2 expression (Figure 6). The results of this study show that α-MG significantly protected ARPE-19 cells against NaIO_3_-induced toxicity, high ROS levels, and apoptosis in vitro. Most importantly, the results of this study, for the first time, support a model whereby α-MG inhibits PI3K/AKT-PGC-1α signaling, which is required for the regulation of SIRT-3 protein expression (Figure 11). 

## 5. Conclusions

In summary, our in vitro and in vivo experimental results showed that α-MG could protect retinal cells (especially RPE cells) from oxidative stress damage via apoptosis. The protective function of α-MG on oxidative stressed RPE cells is expressed through its antiapoptotic effect. As a result, α-MG may be able to serve as a potent therapeutic agent for the treatment of retinal degeneration diseases. 

## Data Availability

Data are contained within the article.
